# Silent Separation: Uterine Dehiscence After Second-Trimester Termination in Monochorionic Diamniotic Twins

**DOI:** 10.7759/cureus.99175

**Published:** 2025-12-14

**Authors:** Ayisha Ejaz Bhutta, Karen E Austin-Smith, Wael Abdulkareem, Nimatallah Omoteso

**Affiliations:** 1 Department of Obstetrics and Gynaecology, Kettering General Hospital, Kettering, GBR; 2 Department of Obstetrics and Gynaecology, Latifa Women and Children Hospital, Dubai, ARE

**Keywords:** bakri balloon, case report, misoprostol, second-trimester induction, twin pregnancy, uterine rupture

## Abstract

Uterine rupture is a rare but life-threatening complication, most commonly associated with labor in women with a prior caesarean scar. Its occurrence in the second trimester, particularly in primigravid women with an unscarred uterus, is exceptionally uncommon. Here, we present a 29-year-old primigravida with a monochorionic-diamniotic (MCDA) twin pregnancy complicated by absent end-diastolic flow in one twin and subsequent intrauterine fetal demise at 21+5 weeks. Induction of labor with misoprostol resulted in delivery at 21+6 weeks. The early postpartum course was complicated by pyrexia, retained tissue, and recurrent heavy bleeding requiring ultrasound-guided surgical evacuation (SEVAC), uterine balloon tamponade, and major obstetric hemorrhage. Thirty-two days post-delivery, the patient collapsed with secondary postpartum hemorrhage. MRI later demonstrated a complete absence of the anterior uterine wall, consistent with rupture/dehiscence. Laparotomy confirmed a large anterior wall defect, which was successfully repaired, preserving fertility. This case gives us insight that second-trimester uterine rupture, though rare, is a potentially devastating condition. Clinicians should remain vigilant regardless of parity or uterine history. “Silent rupture” emphasizes the role of imaging in ambiguous cases. Conservative measures for postpartum hemorrhage must be used judiciously, and prompt surgical management optimizes outcomes and preserves fertility. Larger studies are needed to refine risk stratification and monitoring strategies.

## Introduction

Uterine rupture is a rare obstetric emergency, most often associated with trial of labor after cesarean (TOLAC) or previous uterine surgery [1]. The risk of uterine rupture in women with prior caesarean delivery is 0.28% (95% CI 0.08-1.00%), compared with 0.04% (95% CI 0.01-0.20%) in those without previous caesarean delivery [2]. While rupture at term is well described, its occurrence in the second trimester is far less common and poses significant diagnostic challenges [3].

Medical induction with misoprostol has become standard practice for second-trimester termination of pregnancy, given its effectiveness and safety profile [4]. However, rare cases of uterine rupture have been reported, both in scarred and unscarred uteri, with similar incidence patterns [2,5]. Recent systematic reviews suggest rupture may occur without classic symptoms such as acute abdominal pain or maternal collapse, termed “silent rupture” [3,6].

The use of mechanical and pharmacologic interventions together, such as misoprostol and uterine balloon tamponade, has been linked to uterine injury in certain contexts [7,8]. This underscores the need for caution, particularly in complex pregnancies or when infection is present [9]. We report a case of second-trimester uterine rupture in a primigravida with an unscarred uterus following induction for intrauterine fetal demise in an MCDA twin pregnancy. The case illustrates diagnostic complexity, the value of advanced imaging, and potential risks of adjunctive interventions such as Bakri balloon tamponade in compromised uterine tissue [7].

## Case presentation

A 29-year-old primigravida, booking BMI 24.9, presented with an MCDA twin pregnancy complicated by absent end-diastolic flow (EDF) in Twin B and intermittent growth discordance (~8%) [1]. Low PAPP-A was noted in early screening, and she was identified as a carrier of Group B Streptococcus. Antenatal monitoring for twin-to-twin transfusion syndrome (TTTS) was done, but the criteria for fetoscopic laser were not met.

At 21+5 weeks, intrauterine demise of both fetuses was confirmed. Following compassionate counselling, induction of labor with misoprostol led to vaginal delivery at 21+6 weeks. The guidelines used were the FIGO Mifo-Miso guideline. Estimated blood loss was 245 mL. Postpartum, the patient developed pyrexia, which was treated with intravenous antibiotics.

**Figure 1:**
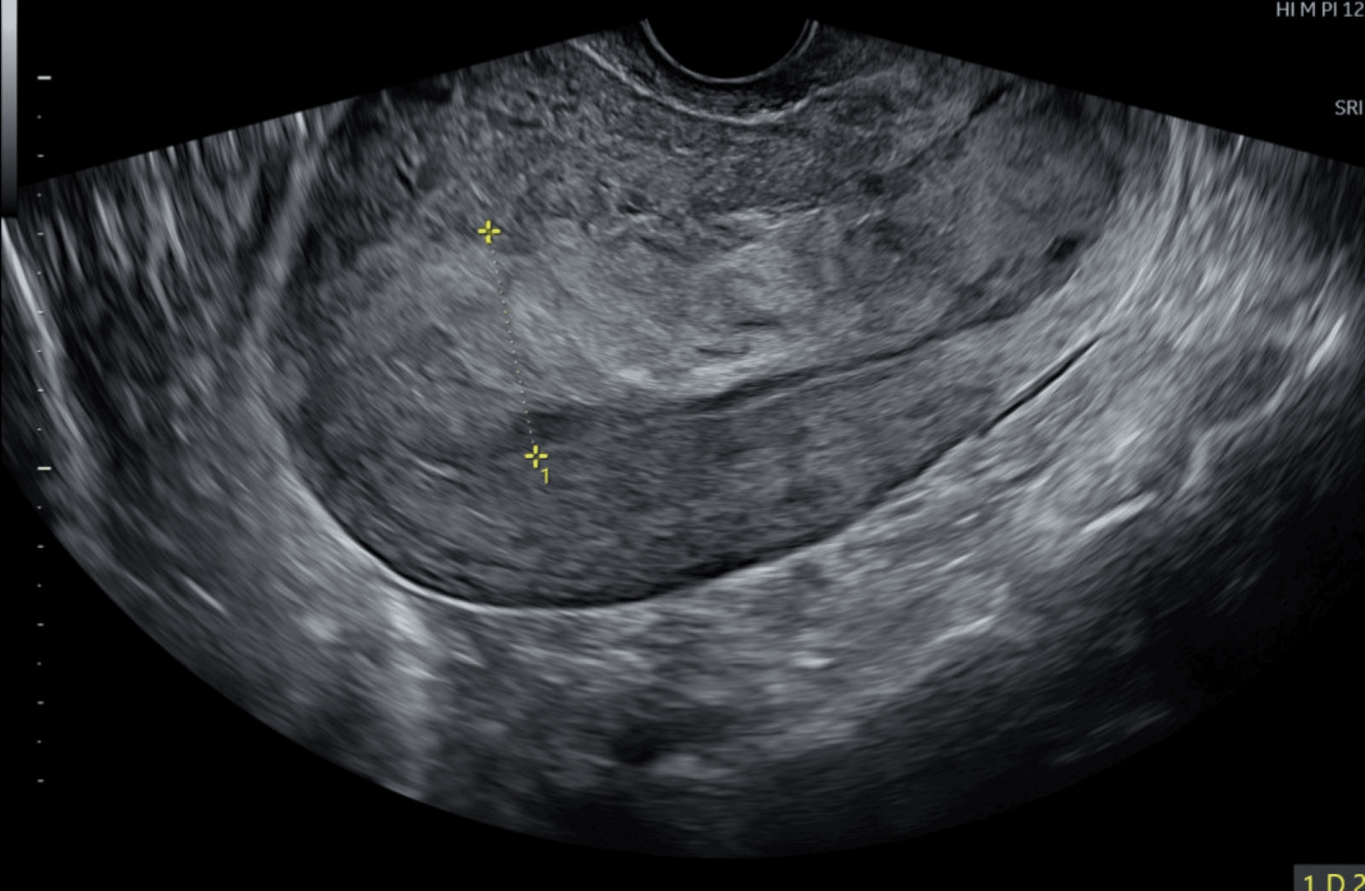
Ultrasound scan image showing the endometrial thickness of 29 mm.

On day 8 postpartum, she presented with passage of clots. Examination revealed tissue protruding through the cervix, which was removed and sent for histopathology. Ultrasound showed thickened endometrium (29 mm) but no large, retained products. She was discharged after clinical improvement (Figure 1). 

On day 32, she presented with profuse vaginal bleeding, collapse, and hemodynamic instability. Ultrasound-guided surgical evacuation (SEVAC) was performed. During the procedure, a massive postpartum hemorrhage (estimated 1.5 L) occurred, managed with uterotonics, tranexamic acid, uterine balloon tamponade, vaginal packing, and transfusion of 2 units of red blood cells, under major obstetric hemorrhage protocol.

**Figure 2:**
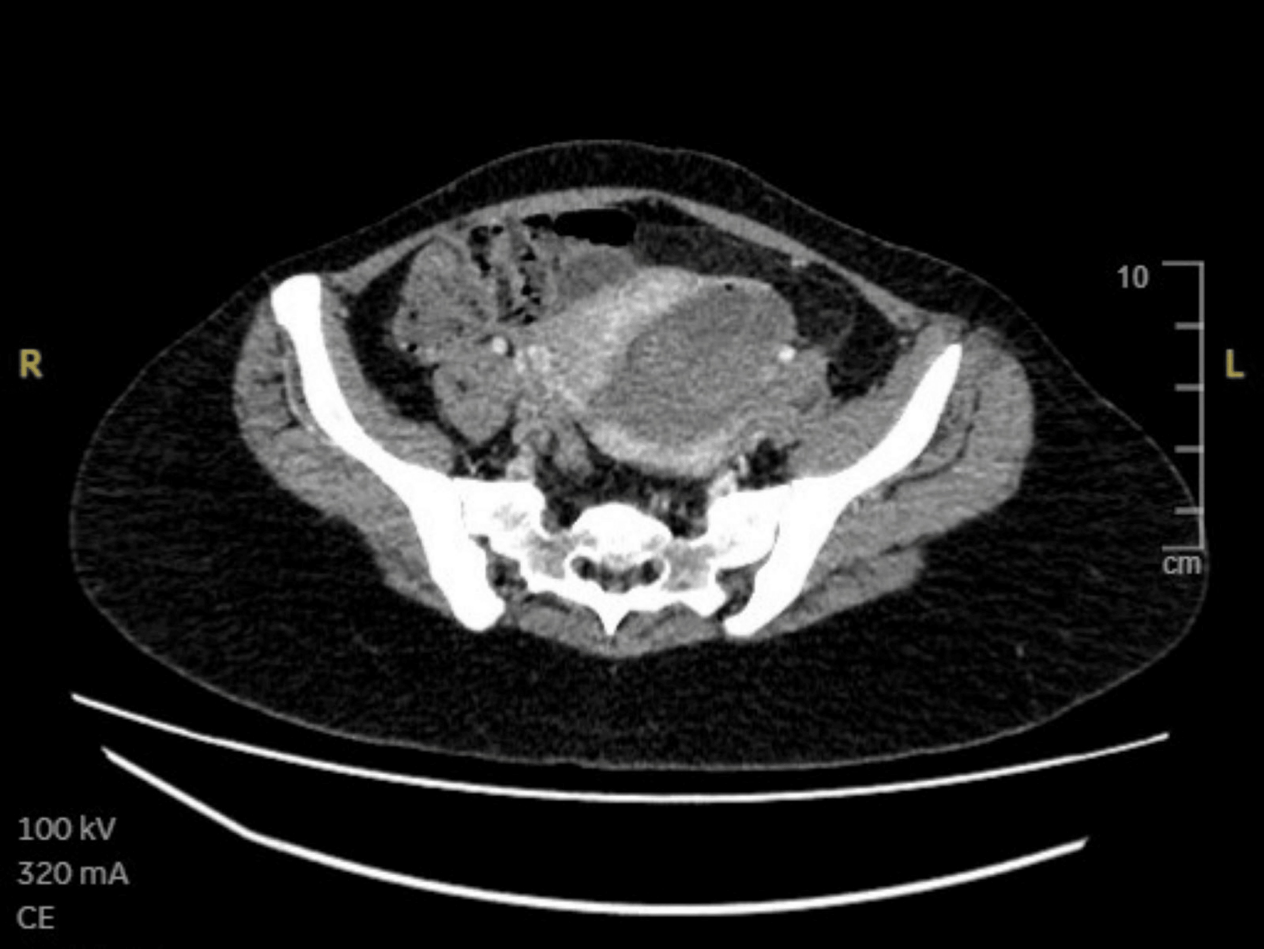
CT scan, which was inconclusive for uterine wall compromise.

**Figure 3:**
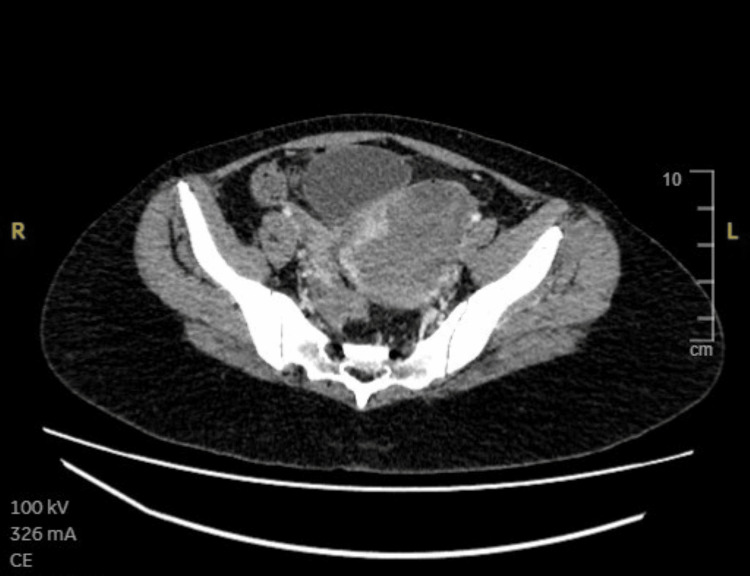
CT scan reported as post-miscarriage for twins. There is an intrauterine collection, containing heterogeneous material in keeping with intraluminal residual hemorrhage.

Despite initial stabilization, intermittent recurrent bleeding occurred. A CT abdomen, pelvis was done, which was reported as showing an intrauterine collection containing heterogeneous material in keeping with intraluminal residual hemorrhage (Figure 2 and Figure 3).

**Figure 4:**
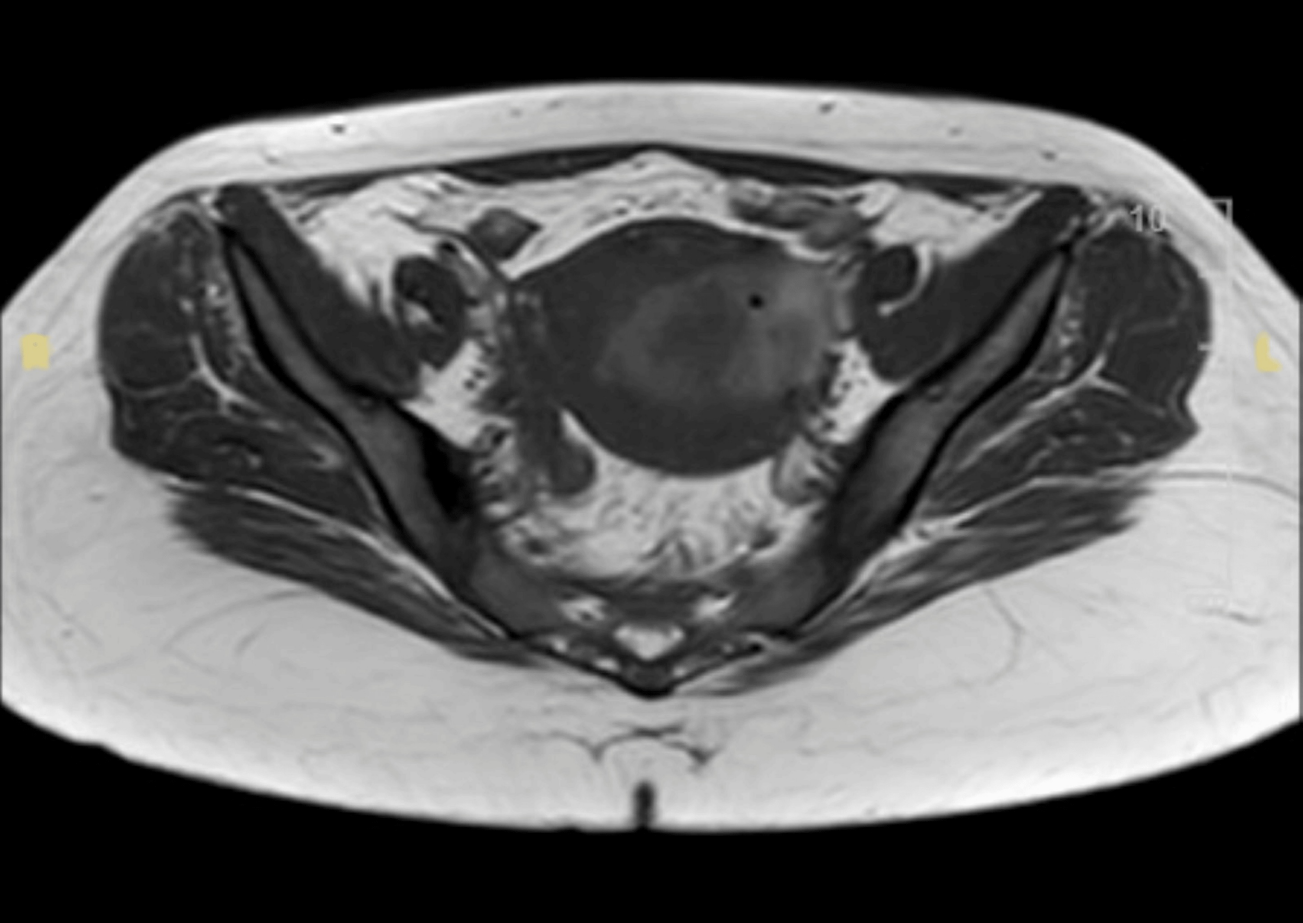
MRI reported as follows: appearances are suspicious of uterine rupture/dehiscence.

**Figure 5:**
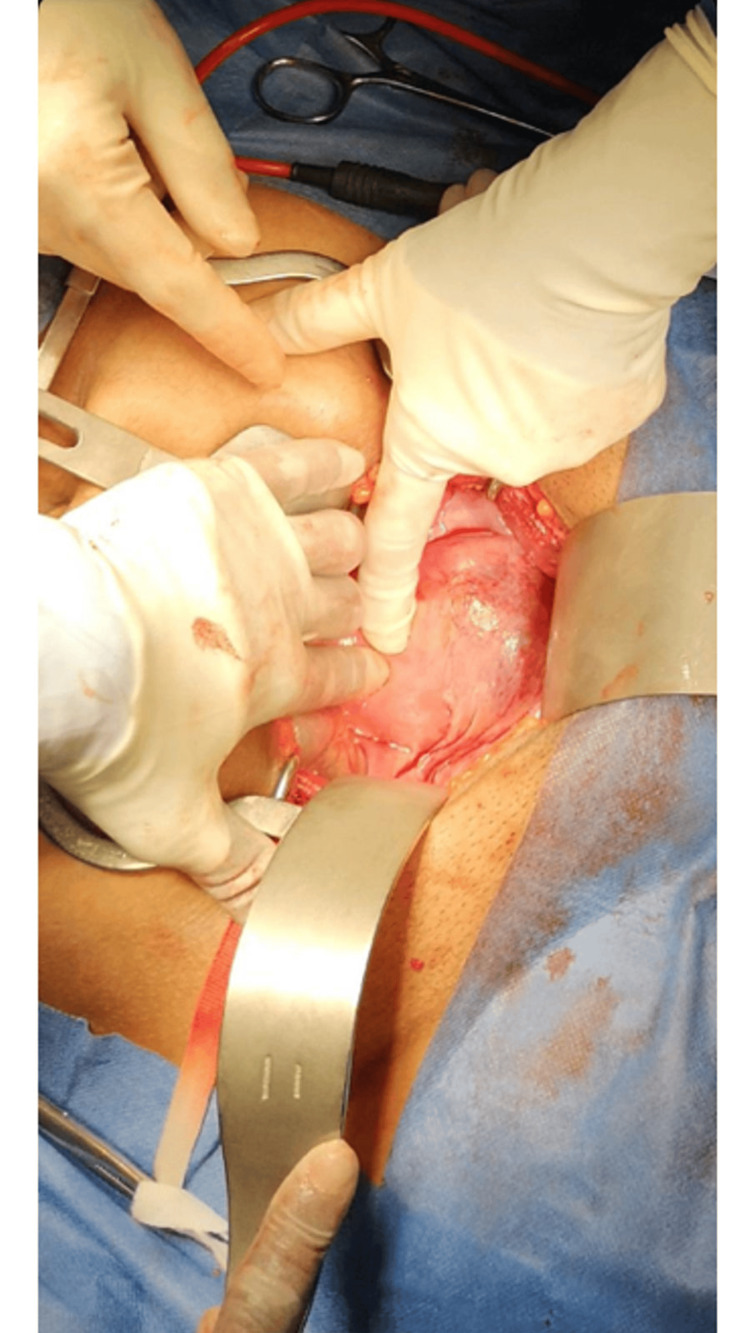
Intra-operative uterine dehiscence repair.

Following a CT scan being inconclusive, an MRI was done on day 41, as shown in Figure 4, demonstrating a complete absence of the anterior uterine wall with a large hematoma, consistent with rupture/dehiscence, which was deemed conclusive. Emergency laparotomy confirmed a large anterior uterine wall dehiscence, with all layers being ruptured except the serosa. The uterine wall was repaired in layers, as shown in Figure 5.

**Figure 6:**
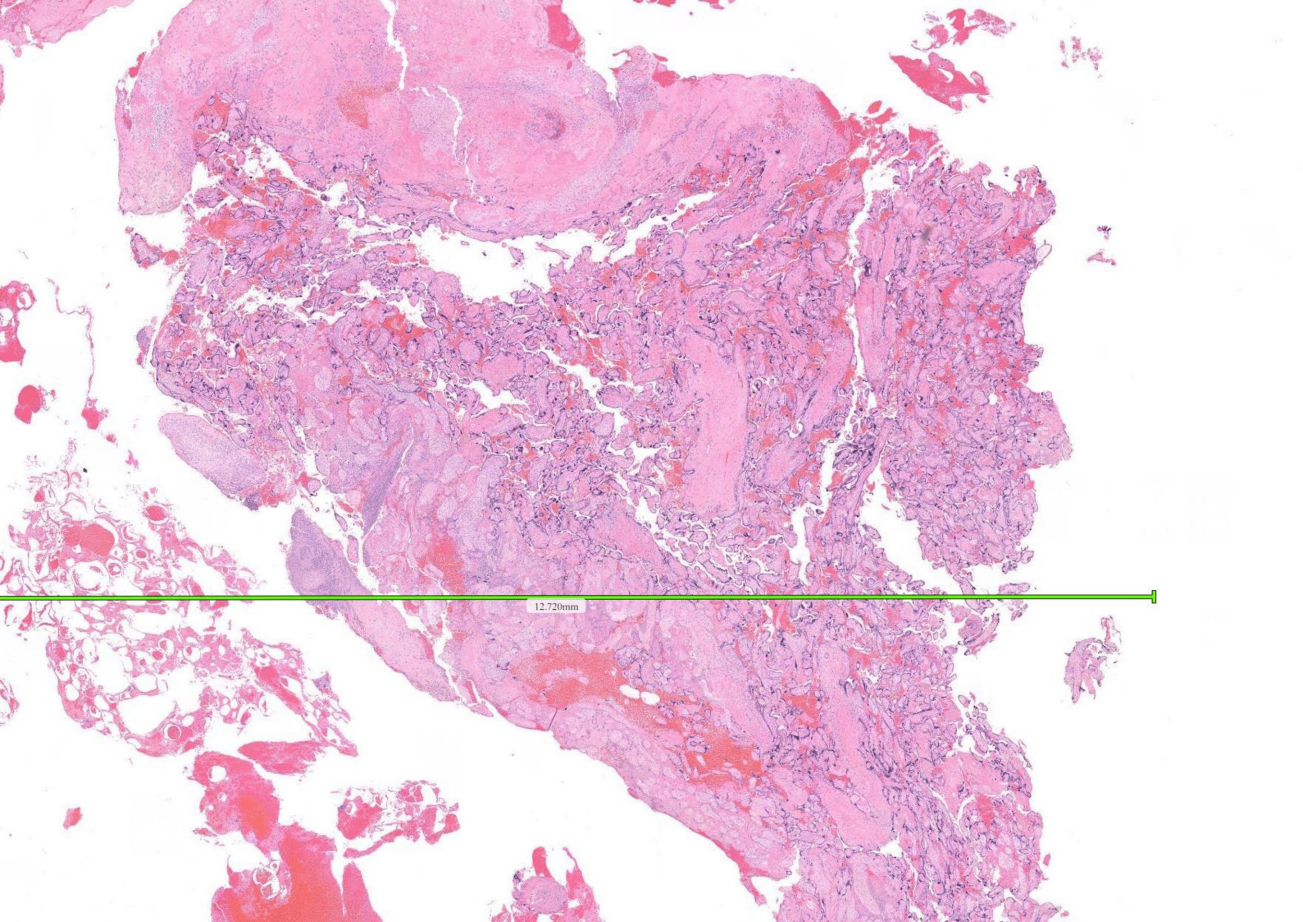
Histology image showing retained products of conception (RPOC) Part 1. These are formalin-fixed tissues, embedded in wax block and sectioned at 3 microns thickness, then stained with hematoxylin and eosin.

**Figure 7:**
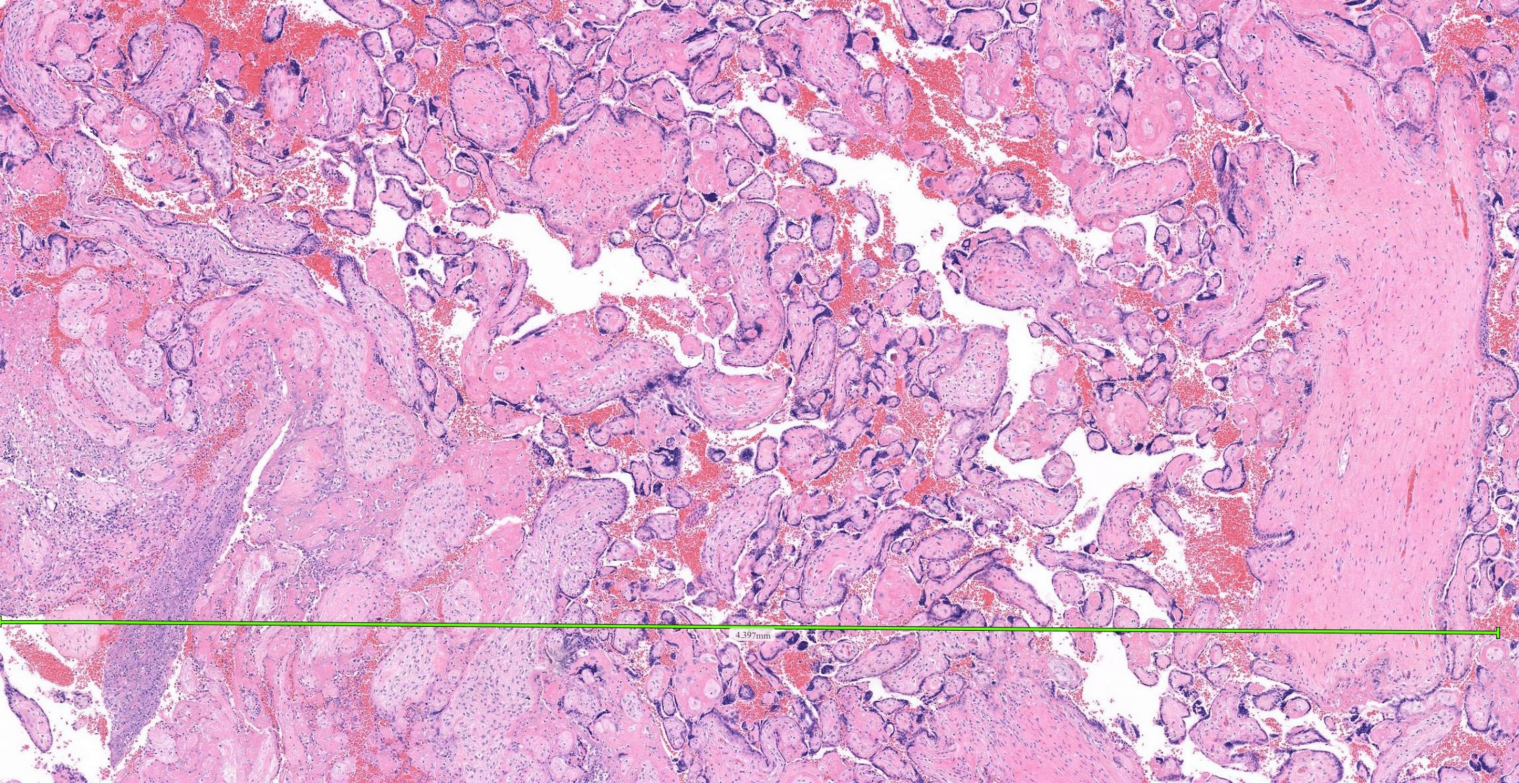
Histology image showing retained products of conception (RPOC) Part 2. These are formalin-fixed tissues, embedded in wax block and sectioned at 3 microns thickness, then stained with hematoxylin and eosin.

Histopathology from prior SEVAC confirmed retained placental tissue (Figure 6 and Figure 7).

Postoperative recovery was uneventful, and the patient was discharged on day 4 with oral antibiotics, iron, and enoxaparin prophylaxis. She was counselled to avoid conception for 12 months and deliver future pregnancies by elective caesarean at 37 weeks.

## Discussion

This case highlights a rare presentation of uterine dehiscence occurring in the second trimester in a primigravida with an unscarred uterus. Multiple interventions, including the administration of misoprostol, SEVAC, and the use of uterine balloon tamponade, may have contributed to the outcome. While it remains challenging to delineate the precise causative factor, the constellation of these procedures underscores the multifactorial nature of the event. Importantly, this case emphasizes the need for heightened vigilance and timely identification of potential risk factors, as well as the judicious use of uterotonic and mechanical interventions to mitigate adverse outcomes.

Several risk factors for uterine rupture were present in this case, including misoprostol use, SEVAC, and uterine balloon tamponade. Although the exact etiology cannot be clearly attributed to one specific factor, the combination of pharmacologic and mechanical interventions likely increased uterine stress and friability. While uterine rupture is classically associated with prior caesarean delivery, it is increasingly reported in unscarred uteri following misoprostol induction [2,4,5,10]. Although the absolute risk remains low, the consequences are severe, warranting vigilance even in patients considered low-risk [2,11].

The presentation was atypical, characterized by recurrent intermittent bleeding, infection, and delayed deterioration, aligning with the “silent rupture” literature [2,5]. MRI was decisive in diagnosing the complete absence of the anterior uterine wall, guiding surgical management [6,12]. Although robust data on perforation risk associated with SEVAC are limited, a single-center study demonstrated that routine intraoperative ultrasound guidance during uterine evacuation significantly reduced perforation rates from 1.4% to 0.2% [13]. This finding highlights the importance of intraoperative imaging to enhance procedural safety.

Uterine balloon tamponade, although effective for postpartum hemorrhage, may exacerbate pre-existing uterine compromise [3,8,9]. Therefore, it should be used cautiously, particularly in the context of infection or tissue friability. Early laparotomy and surgical repair have been shown to preserve fertility when rupture is promptly recognized and managed [7,14,15]. A report by Ajayi et al. described sequential uterine curettage and balloon tamponade contributing to rupture, emphasizing the need for careful clinical judgment [16]. Thorough pre-procedure counselling before second-trimester induction, particularly in twin pregnancies or those complicated by infection, is essential [2,5]. Subsequent pregnancies following conservative repair, including laparoscopic techniques, have demonstrated favorable outcomes [17].

This case underscores the importance of considering uterine rupture as a differential diagnosis following second-trimester induction, even in primigravid patients with unscarred uteri [2,5]. Persistent or unexplained bleeding after induction warrants advanced imaging, particularly MRI, to exclude occult dehiscence or rupture [6,12]. Finally, clinicians must remain mindful that interventions such as SEVAC and Bakri balloon tamponade, although lifesaving in many scenarios, carry potential risks in both compromised and unscarred uteri [3,8,9]. Early recognition and timely surgical repair remain pivotal to optimizing outcomes and preserving fertility [7,14-17].

## Conclusions

In this case, MRI played an important role when ultrasound and CT were insufficient to clarify the diagnosis, offering superior soft-tissue characterization without radiation exposure. Counseling for second-trimester SEVAC emphasized reassuring the patient that the procedure aims to safely manage retained tissue and reduce the risks of infection or significant bleeding while supporting overall well-being. Discussions also addressed general surgical and anesthesia-related risks, such as hemorrhage, infection, cervical trauma, uterine injury, and the rare possibility of repeat evacuation or unplanned laparotomy, along with options for pain management, emotional and bereavement support, and follow-up to monitor recovery and future reproductive health. This case highlights that second-trimester uterine rupture, though rare, is potentially devastating, underscoring the need for clinical vigilance regardless of parity or uterine history. The concept of “silent rupture” reinforces the value of appropriate imaging in ambiguous presentations. Conservative measures for postpartum hemorrhage should be applied judiciously, while timely surgical management remains essential for optimizing outcomes and preserving fertility. Larger studies are needed to refine risk stratification and guide monitoring strategies in such uncommon but high-impact clinical scenarios.
